# MT1X is an oncogene and indicates prognosis in ccRCC

**DOI:** 10.1042/BSR20221128

**Published:** 2022-10-18

**Authors:** Yanpeng Ding, Jiayu Fang, Mengge Chen, Yulian Xu, Nuomin Liu, Sha Fang, Wenbin Xiang, Rui Chen, Chaoyan Wu, Haijun Yu

**Affiliations:** 1 Department of Radiation and Medical Oncology, Hubei Key Laboratory of Tumor Biological Behaviors, Hubei Cancer Clinical Study Center, Zhongnan Hospital of Wuhan University, Wuhan 430071, China; 2 Xiangyang Central Hospital, Affiliated Hospital of Hubei University of Arts and Science, Xiangyang 441000, China; 3 Department of Otorhinolaryngology-Head and Neck Surgery, Zhongnan Hospital of Wuhan University, Wuhan 430071, China; 4 Department of Radiation Oncology, Huadong Hospital Affiliated to Fudan University, Shanghai 200040, China; 5 Department of Oncology, First People’s Hospital of Zaoyang, Zaoyang 441200, China; 6 Department of Hepatobiliary Surgery, The First Affiliated Hospital of Xi’an Jiaotong University, Xi’an 710000, China; 7 Department of Traditional Chinese Medicine, Zhongnan Hospital of Wuhan University, Wuhan 430030, China

**Keywords:** biomarker, clear cell renal cell carcinoma, immune infiltration, MT1X, prognosis

## Abstract

The metallothionein 1 (MT1) family was previously shown to be involved in metal ion homeostasis, DNA damage, oxidative stress, and carcinogenesis. Our team’s previous study showed that MT1X is most closely associated with ccRCC. However, its role in clear cell RCC (ccRCC) remains unclear. The present study aimed to demonstrate MT1X’s prognostic value, potential biologic function, impact on the immune system, and influence on cell growth, the cell cycle, apoptosis, and migration in the setting of ccRCC. The relationship between clinical pathologic features and MT1X was analyzed using bioinformatics. We knocked down MT1X in the ccRCC cell line 786O with si-MT1X to verify the results of the bioinformatic analysis at the cytological level. Apoptosis assay, cell cycle assay, wound-healing assay, colony formation assay, and RT-qPCR were performed. MT1X is correlated with the stage (T and M) and grade and is able to be an independent prognostic factor for ccRCC. The TISIDB database analysis showed a significant correlation between MT1X and tumor-infiltrating lymphocytes such as central memory CD8^+^ T cells and γΔT cells. MT1X was also positively related to immunomodulators such as TGFB1 and CXCR4. We also found that MT1X knockdown inhibits cell growth, induces apoptosis, arrests cells in the S cell cycle, and inhibits the wound healing proportion in ccRCC. Gene set enrichment analysis and quantitative PCR (q-PCR) analysis found that down-regulation of MT1X reduced the accumulation of hypoxia-associated factors. Bioinformatic analysis associated increased MT1X expression with a worse prognosis. Laboratory experiments confirmed bioinformatic findings. MT1X was also found to be an independent prognostic biomarker for ccRCC and is involved in immune system regulation.

## Introduction

Renal cell carcinoma (RCC) is the most common cancer derived from the kidney. RCC is classified into various subtypes. Based on the 2018 global cancer statistics, approximately 403,262 kidney cancer cases are newly diagnosed, and approximately 175,098 patients died of RCC [1]. Tobacco, obesity, hypertension, and chronic kidney disease history are proven risk factors for RCC [2]. Exposure to cadmium and cadmium-containing compounds has also been shown to increase the risk of RCC [3]. Clear cell RCC (ccRCC), the most popular subtype of RCC that originates from the epithelium of the proximal tubule of the nephron [4], accounts for approximately 85% of RCC tumors [5]. Although ccRCC can be cured via surgical resection, patients diagnosed early benefit most from surgery [6]. Up to 30% of patients diagnosed with ccRCC already have distant metastases [7]. Compared with chemoradiotherapy, molecule-targeted drugs such as sorafenib and sunitinib have greatly improved the overall survival (OS) of ccRCC patients [8]. However, the side effects and variable patient response to these treatments have led to a low OS rate among ccRCC patients [9]. The underlying molecular mechanisms of ccRCC are unclear. It, therefore, makes sense to explore the disease mechanisms behind ccRCC in the hope of discovering new factors or potential biomarkers related to its prognosis.

MT1X belongs to the metallothionein (MT) family, a group of cadmium binding and low molecular weight proteins first discovered in 1957 [10]. MTs are a group of cysteine-rich intracellular proteins with four defined subtypes (MT1–MT4) and weights ranging from 6 to 7 kDa [11]. MTs play a crucial role in metal ion homeostasis and detoxification [12]. Their high chemical attraction to heavy metals enables them to bind to xenobiotic heavy metals and protect cells from metal toxicity [13]. Prior evidence have shown that MT can regulate cell growth and proliferation, protect cells against oxidative stress, serve as antineoplastic drugs, and reduce the effects of radiation by binding to heavy metals such as zinc/copper [14,15]. An increasing body of evidence has shown that MTs play a role in tumor progression, metastasis, and drug resistance and can act as protectors against DNA damage and apoptosis [10,16,17]. The expression of MTs differs between different cancers and can affect the type and differentiation of tumors and environmental incitant and gene mutations [18].

This is the first study to evaluate the clinical value of MT1X in ccRCC. The expression level, prognosis, effect on ccRCC progression *in vitro*, interaction with the host immune system, and potential regulatory mechanisms of MT1X in ccRCC have not been previously described. Here, we performed bioinformatic analysis and laboratory experiments to demonstrate the potential clinical and biological value of MT1X in ccRCC.

## Materials and methods

### Data acquisition

The clinical information and MT1X expression of patients with ccRCC were downloaded from the TCGA database [19]. Our study included 495 ccRCC samples and excluded any samples with missing or insufficient data on TNM, disease stage, or tumor grade. RNA sequences and clinical information were preserved and used for further analysis.

### Oncomine

Oncomine integrates previously published GEO, TCGA, and RNA-seq data. We used it to quantify the MT1X expression of different human cancers (https://www.oncomine.org/resource/main.html) [20]. Thresholds were set at *P*-value = 1E-4, fold change = 2, gene rank = top 10%, and the data type must be mRNA.

### TIMER

TIMER is an online database that includes 10,897 samples of 32 different human cancers from the TCGA database (https://cistrome.shinyapps.io/timer/) [21]. We used the Wilcoxon test to confirm the different expression levels of MT1X between the tumor and adjacent normal tissue.

### Kaplan–Meier plotter

The correlation between MT1X and OS in ccRCC was analyzed using Kaplan–Meier plotter. Kaplan–Meier plotter is an extensive database that contains GEO, EGA, and TCGA, embracing gene expression and clinical data. Patient samples were classified into high expression and low expression cohorts to assess the prognostic value of MT1X. It is a reliable tool to evaluate the correlation between gene expression and survival in 21 cancer types [22] (www.kmplot.com).

### Cell lines and cell culture

Human VHL defective ccRCC cell line 786O originated from the primary tumor tissue, remained in RPMI (Gibco, U.S.A.) supplemented with 10% fetal bovine serum (Gibco, U.S.A.) at 37°C and 5% CO_2_. Hypoxic conditions were simulated using a hypoxia chamber (Thermo, U.S.A.) containing 1% O_2_, 5% CO_2_, and 94% N_2_ at 37°C. Cells were cultured in the hypoxia chamber for 48 h and then prepared for RT-qPCR analysis for hypoxia experiments.

### Apoptosis and cell cycle assay

Cells were transfected with si-MT1X (Qijing, China) or si-control (786O-si-1/2 or 786O-si-Ctrl) for 48 h, then collected for either double staining with Annexin V-FITC/propidium iodide (PI) or single staining with PI according to the manufacturer’s protocol (Annexin V-FITC/PI Apoptosis Kit, Cell cycle staining kit; MultiSciences, China). Flow cytometry (Beckman, U.S.A.) was used to measure the percentage of stained cells. Experiments were performed in triplicate. The target sequences of si-MT1X were provided in Supplementary Table S1. Experiments were performed in triplicate, and experimental comparisons between the si-MT1X and control groups were analyzed using Student’s *t*-test by GraphPad Prism 8.

### Wound-healing assay

Cell were transfected with si-RNA for 24 h, then seeded into six-well plates with a density of approximately 95% confluence after 24 h. The monolayer was lightly scratched with a 200-μl pipette tip across the well. After scratching, the well was washed with PBS and then replenished with serum-free medium for 12 h. Experiments were performed in triplicate, and Student’s *t*-test was used to analyzed si-group and control group.

### Colony formation assay

A colony formation assay was used to analyze the effect of MT1X on cell growth. Both 786O-siMT1X (786O-si-1 or 786O-si-2) and 786O-Ctrl transfected cells were seeded into six-well plates (200 cells/well). After 10 days, the colonies were fixed with 4% paraformaldehyde (PFA) and stained with crystal violet. The rate of colony formation was calculated using the following formula: (number of colonies formed/number of inoculated cells) × 100%. Experiments were performed in triplicate, and Student’s *t*-test was used.

### Quantitative PCR (q-PCR) and RNA isolation

Total RNA was isolated from 786O-si-1 and 786O-si-Ctrl cells using the RNA extraction Kit (Aidlab, China), according to the manufacturer’s protocol. After measuring the total RNA concentration, cDNA was generated using the HiScript® Q RT SuperMix (Vazyme, China). ChamQ SYBR qPCR Mix (Vazyme, China) was used for quantitative analysis. Primers used in this study were provided in Supplementary Table S2. Repeat experimental method and analysis method are the same as above.

### TISIDB

TISIDB (http://cis.hku.hk/TISIDB) is a comprehensive database containing 988 immune-related antitumor genes, high-throughput screening techniques, genomic profiling data, and immunological data from seven public databases [23]. The TISIDB database was used in the present work to analyze the interactions between MT1X and lymphocytes with immunomodulators.

### Gene set enrichment analysis

To study the biological functions of MT1X, gene set enrichment analysis (GSEA) was used to analyze normalized RNA-seq data obtained from the TCGA database. GO terms and pathway enrichment analysis were performed using GSEA [24]. The number of permutations was set to 1000. Markers of interest had a *P*-value <0.05 and an enrichment score (NES) >1.5.

### Statistical analysis

Patient clinical data were downloaded from TCGA and analyzed using R-4.0.2. Both univariate and multivariate Cox analysis models were used for prognostic analysis. The correlation between clinical characteristics and MT1X expression was analyzed using logistic regression, with a cut-off criterion of a *P*-value < 0.05. A correlation heatmap was used to detect correlations between 22 types of immune cells. Experiments were performed in triplicate, and experimental comparisons between the si-MT1X and control groups were analyzed using Student’s *t*-test by GraphPad Prism 8 (LaJolla, CA, U.S.A.) software.

## Results

### The mRNA expression of MT1X in different types of cancers

**Figure 1:**
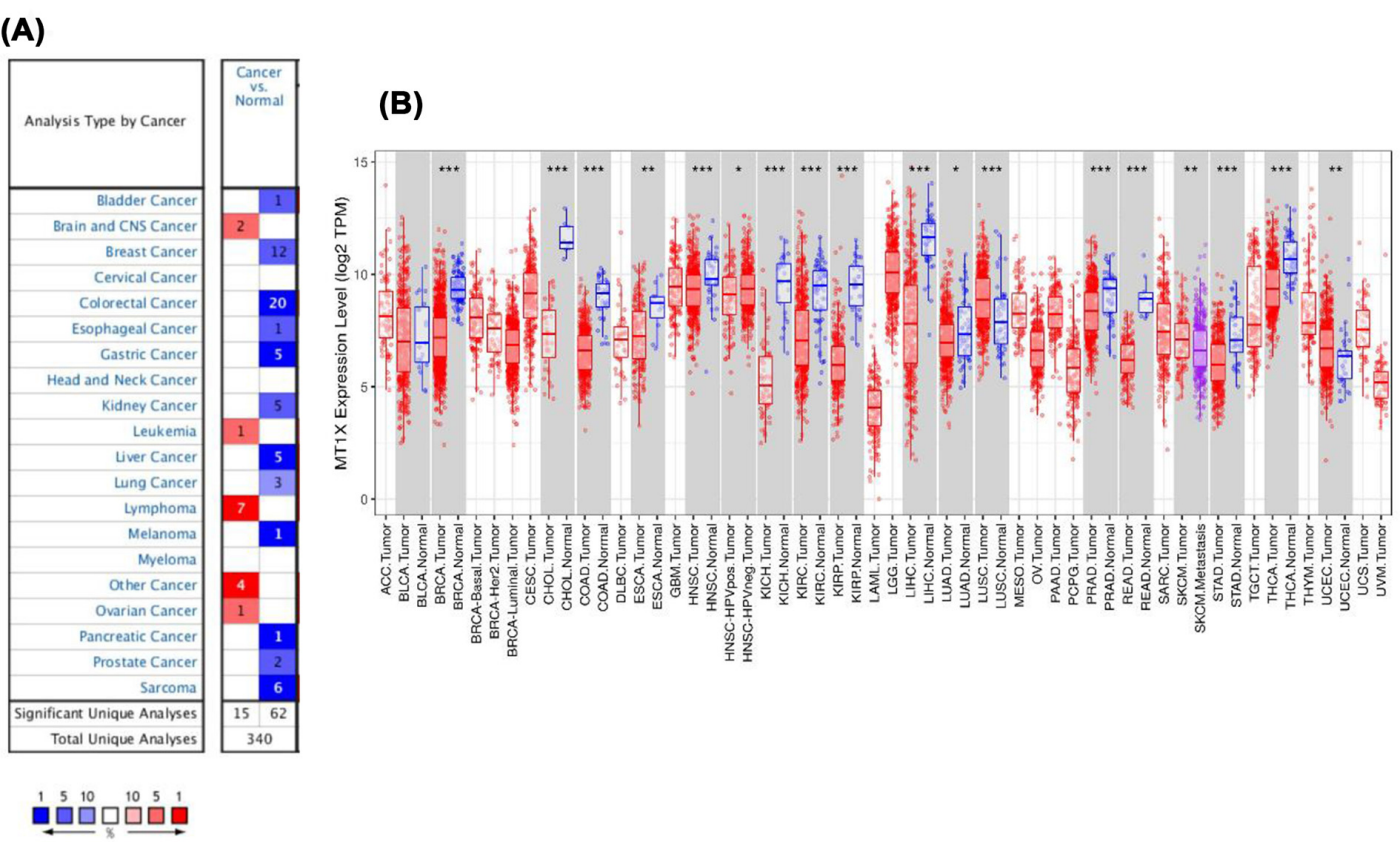
MT1X expression levels in different types of human cancers (**A**) Oncomine database. The numbers in the colored squares represent the number of involved studies. Different colors represent different expression levels of MT1X in those studies; red represents high expression, and blue represents low expression. The darker the red color, the higher expression is. The darker the blue color, the lower the expression is. (**B**) TCGA database detected by TIMER. The red color box represents the tumor tissues, the blue box represents the normal tissue and the purple box represents the metastasis tissue (*P<0.05, **P<0.01, ***P<0.001).

Oncomine was used to identify differences in MT1X expression in tumor tissues compared with normal tissues, result shown in Figure 1A that MT1X was down-regulated in 12 human cancers and up-regulated in 5 human cancers. We then analyzed the TCGA database using TIMER based on RNA-seq data (Figure 1B). Results showed a significant difference in expression level between normal tissues and tumor tissues in 17 human cancers (kidney renal clear cell carcinoma was included), which can distinguish normal tissue from tumor tissue.

### Relationship between MT1X and ccRCC clinical characteristics

**Figure 2:**
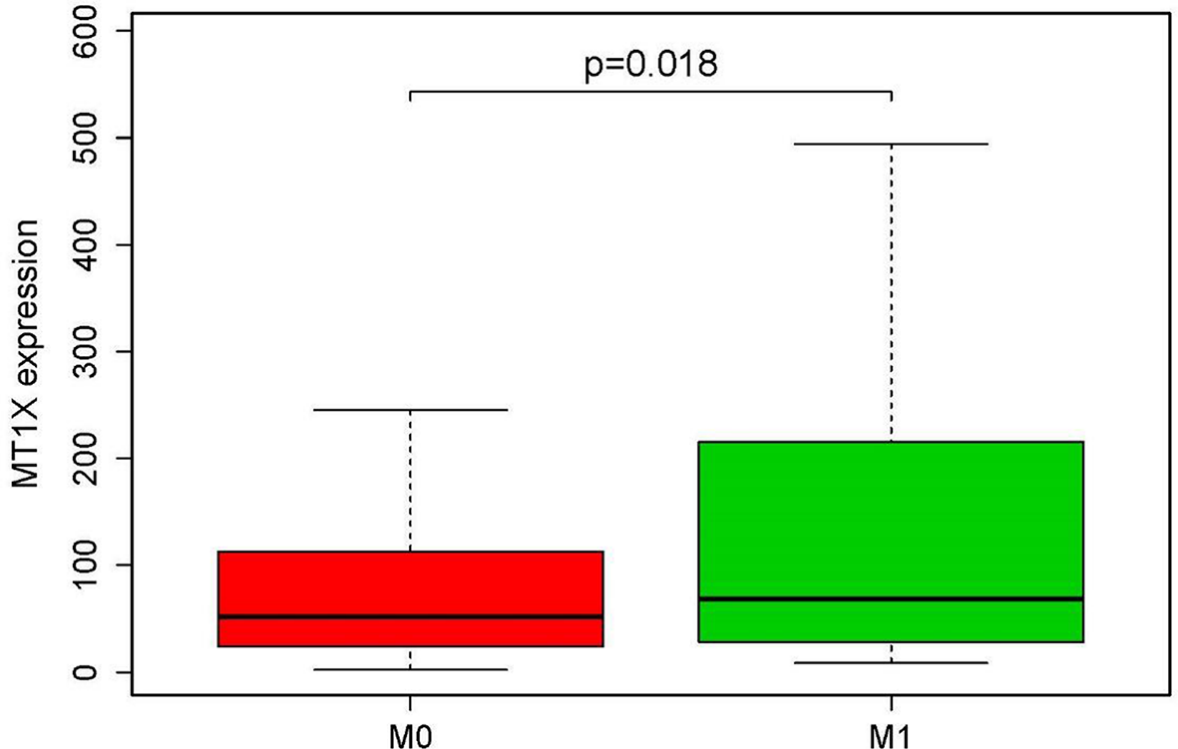
Different MT1X expression levels in different metastatic statuses, tested by the Kruskal test

Using logistic regression (Table 1), MT1X expression conspicuously correlated with patient age (*P*=0.0156), T (T1 vs. T4, *P*=0.0487), grade (II vs. IV, *P*=0.0145; III vs. IV, *P*=0.0005), primary tumor laterality (*P*=0.0432) and person neoplasm status (*P*=0.0008). Due to space considerations, factors with a *P*-value >0.05 were not presented. We further found that high MT1X expression is related to distant tumor metastasis (Figure 2). All results above suggest that ccRCC patients with high expression of MT1X are more liable to have tumors in high tumor stage and grade and more likely to have distant metastasis.

**Table 1:** Association between MT1X expression and clinicopathologic characteristics using logistic regression

Clinical characteristics	*N*	Odds ratio	*P*-Value
Age	495	1.02 (1.00–1.03)	0.0156*
Gender	495	1.18 (0.82–1.72)	0.3762
Stage (I vs. IV)	323	1.61 (0.97–2.71)	0.0671
T (T1 vs. T4)	258	4.76 (1.20–31.65)	0.0487*
Grade			
II vs. IV	289	1.96 (1.15–3.41)	0.0145*
III vs. IV	272	2.72 (1.56–4.84)	0.0005**
Primary tumor laterality	495	0.69 (0.49–0.99)	0.0432*
Person neoplasm status	462	2.02 (1.34–3.05)	0.0008**

(*P<0.05, **P<0.01, ***P<0.001).

### Patient characteristics and multivariate analysis

**Figure 3:**
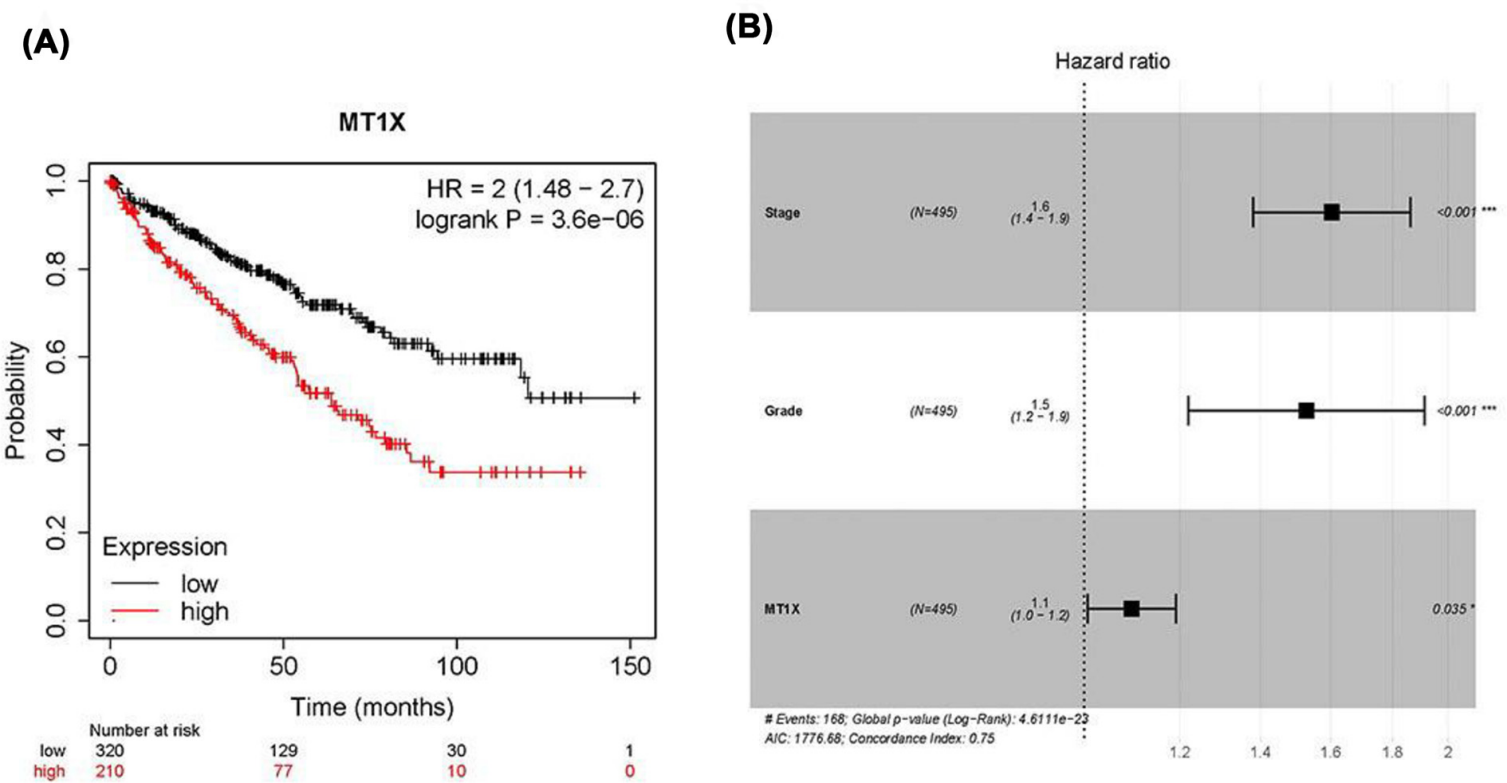
Correlation of MT1X expression with OS and clinicopathologic features (**A**) OS analyzed by Kaplan–Meier plotter (HR = 2, *P*<0.001). (**B**) Multivariate Cox analysis of clinicopathological variables. (*P<0.05, **P<0.01, ***P<0.001).

We evaluated MT1X with Kaplan–Meier plotter and found that increased expression of MT1X in ccRCC predicts unfavorable OS (*n*=530, HR [hazard ratio] = 2, *P*<0.001) (Figure 3A). We also performed a univariate and multivariate analysis using Cox regression to define the relationship between MT1X and OS. The univariate analysis (Table 2A) showed that age, stage, T, M, grade, primary tumor laterality, person neoplasm status, and MT1X expression level were associated with prognosis. Results of the multivariate analysis are shown in Table 2B and as a forest plot (Figure 3B) that suggests the independent prognostic value of MT1X in ccRCC along with stage and grade. Factors with a *P*-value > 0.05 are not all shown in the table.

**Table 2:** Association between MT1X with clinicopathologic characteristics in TCGA patients via (**a**) Cox regression. (**b**) Multivariate survival model

ID	HR	HR.95H	HR.95L	*P*-value
(a)				
Age	1.029	1.042	1.016	1.15E-05***
Gender	1.054	1.443	0.770	0.742
Stage	1.862	2.128	1.629	7.36E-20***
T	1.898	2.239	1.608	3.55E-14***
M	4.324	5.913	3.162	4.82E-20***
Grade	2.265	2.779	1.846	4.82E-15***
Neoadjuvant therapy before resection	2.116	4.012	1.116	0.022*
Primary tumor laterality	0.703	0.953	0.519	0.023*
Person neoplasm status	5.399	7.504	3.885	1.01E-23***
MT1X	1.197	1.304	1.100	3.37E-05***
(b)				
Stage	1.603	1.863	1.380	6.60E-10***
Grade	1.528	1.914	1.220	0.0002**
MT1X	1.095	1.191	1.006	0.0349*

(*P<0.05, **P<0.01, ***P<0.001).

### Clone formation and apoptosis

**Figure 4:**
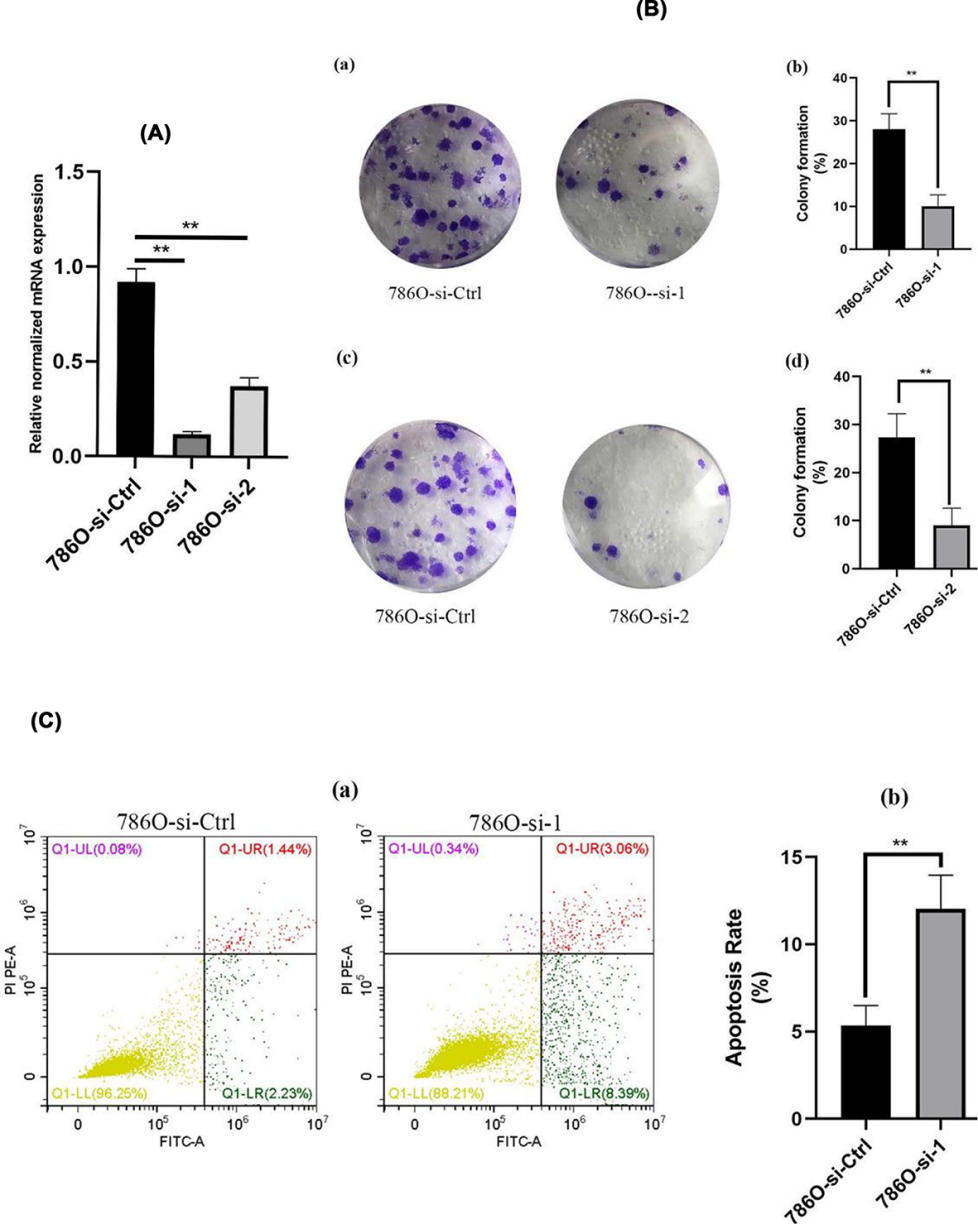
Correlation of MT1X with cell proliferation and apoptosis (**A**) The knockdown of MT1X. (**B**) Knockdown of MT1X inhibits clone formation. (a,c) Clone formation results. (b,d) Statistical analysis (*P<0.05, **P<0.01, ***P<0.001). (**C**) Knockdown of MT1X induces cell apoptosis. (a) Apoptosis results. (b) Statistical analysis (***P<0.001).

Results above demonstrated that overexpression of MT1X contributes to the progression of ccRCC, so we knocked down MT1X to confirm down-regulation of MT1X could inhibit the progression of ccRCC cells. MT1X was significantly down-regulated in 786O-transfected cells with si-MT1X (786O-si-1 and 786O-si-2) compared with cells infected with control siRNA (786O-si-Ctrl) (Figure 4A). As shown in Figure 4B(a,c), the number of clones in the 786O-si-Ctrl group was greater than in the 786O-si-MT1X group (*P*<0.05). Here we chose the 786O-si-1 to perform the apoptosis assay. The apoptosis proportion (Figure 4C) was also greater in the 786O-si-MT1X group (*P*<0.05). Findings suggest that knockdown MT1X inhibits the growth and induces apoptosis in ccRCC cells, consistent with the bioinformatics results above.

### Cell cycle and cell migration assay

**Figure 5:**
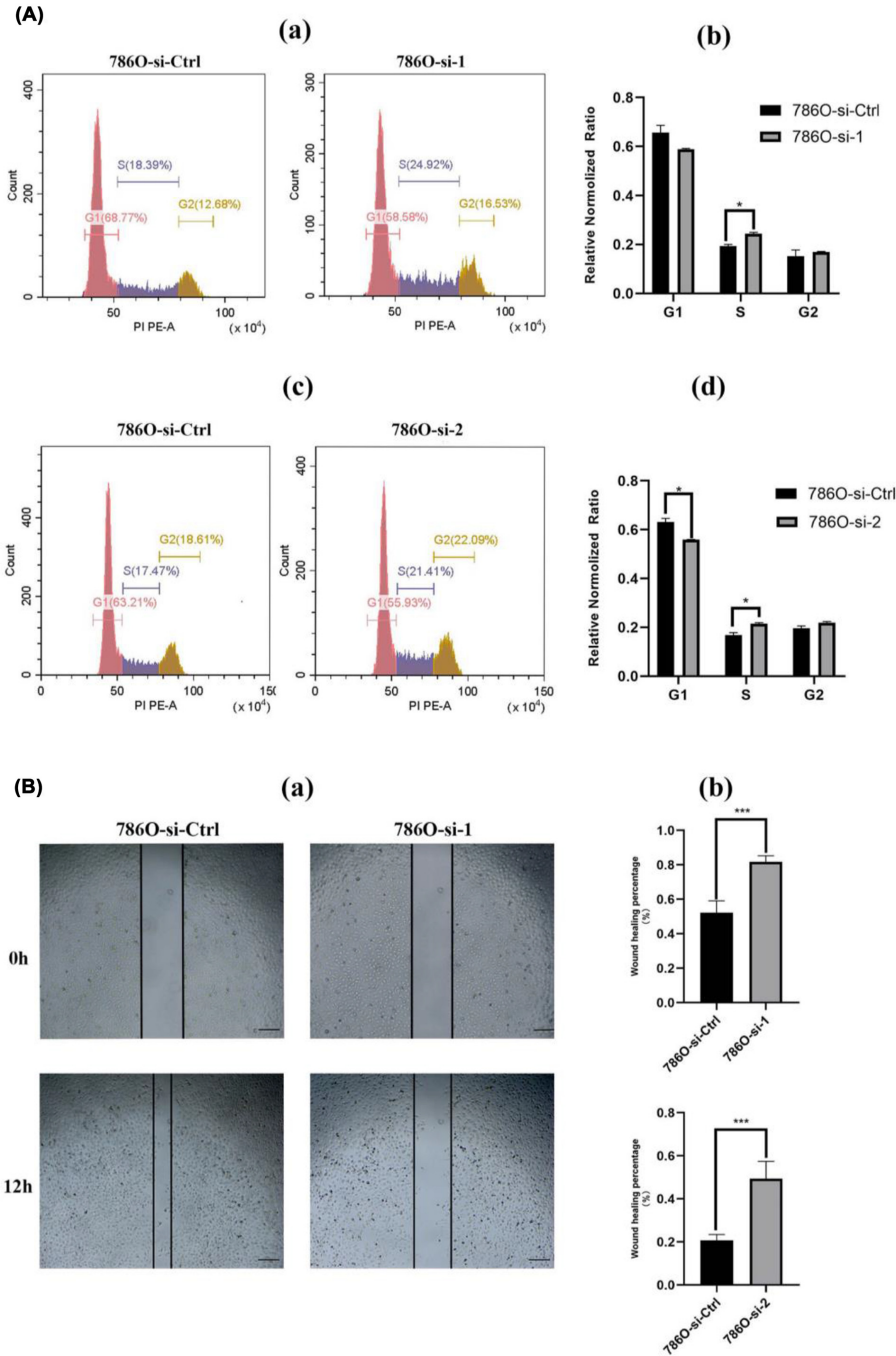
Correlation of MT1X with cell cycle and migration (**A**) Knockdown of MT1X arrests cells in the G1/S cell cycle. (a,c) Cell cycle results. (b,d) Statistical analysis (*P<0.05). (**B**) Knockdown of MT1X inhibits cell migration. (a) Wound healing results of 786O-si-1 vs. 786O-si-Ctrl group (b) Statistical analysis of 786O-si-1/2 vs. 786O-si-Ctrl group (***P<0.001).

We performed cell cycle assay and wound-healing assay. Compared with 786O-si-Ctrl, 786O-si-1 was more likely to be arrested in the S phase (*P*<0.05) and less likely to be in the G1 or G2 phase (*P*>0.05); however, 786O-si-2 was more likely to be arrested in the G1/S phase (Figure 5A). The wound-healing assay results are shown in Figure 5B. After 12 h, the healing distance in the 786O-si-MT1X group was wider than in the 786O-si-Ctrl group, which agrees with the bioinformatic results shown in Figure 3, MT1X knockdown dramatically inhibits the migration ability of ccRCC cells.

### Immune molecules

**Figure 6:**
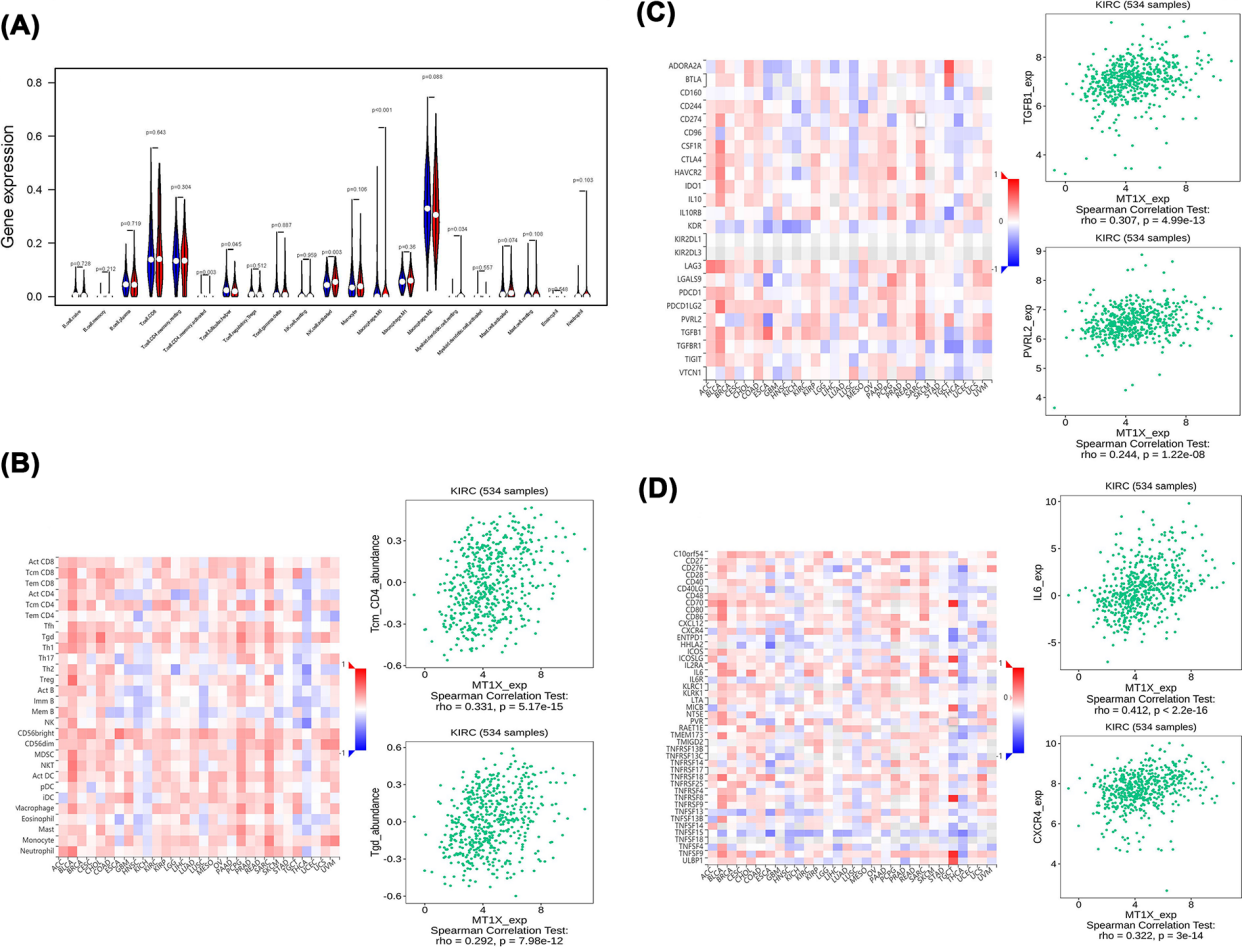
Correlation between MT1X and immune system (**A**) The varied proportions of 22 subtypes of immune cells in high and low MT1X expression groups in tumor groups. (**B–D**) Relationship among the abundance of TILs (B), immunoinhibitors (C), and immunostimulators (D) with MT1X expression, with the top 2 of them displaying the most significant Spearman’s scores.

As shown in Figure 6A, CD4 memory T cells activation, T follicular helper cells, NK cell activation, macrophages MO, and myeloid dendritic cell resting were affected by MT1X expression levels. We then studied the spearman interaction between lymphocytes and immunomodulators and MT1X expression using the TISIDB database. Results showed a significant connection between MT1X expression and tumor-infiltrating lymphocytes (TILs) (Figure 6B), including central memory CD8 T cells (Tcm-CD8) (ρ = 0.274, *P*<0.001), central memory CD4 T cells (Tcm-CD4) (ρ = 0.331, *P*<0.001), γΔ T cells (Tgd) (ρ = 0.292, *P*<0.001), and macrophages (ρ = 0.284, *P*<0.001). Immunomodulators can be further classified into immunoinhibitors, immunostimulators, and major histocompatibility complex molecules. With respect to immunoinhibitors (Figure 6C), MT1X exhibited significantly positive relationships with PVRL2 (ρ = 0.244, *P*<0.001) and TGFB1 (ρ = 0.307, *P*<0.001). With respect to immunostimulators (Figure 6D), MT1X showed a strong correlation with CXCR4 (ρ = 0.322, *P*<0.001), IL6 (ρ = 0.412, *P*<0,001), and PVR (ρ = 0.316, *P*<0.001). MT1X is therefore closely related to the immune microenvironment and regulates immune cells in the setting of ccRCC.

### Gene set enrichment analysis

**Figure 7:**
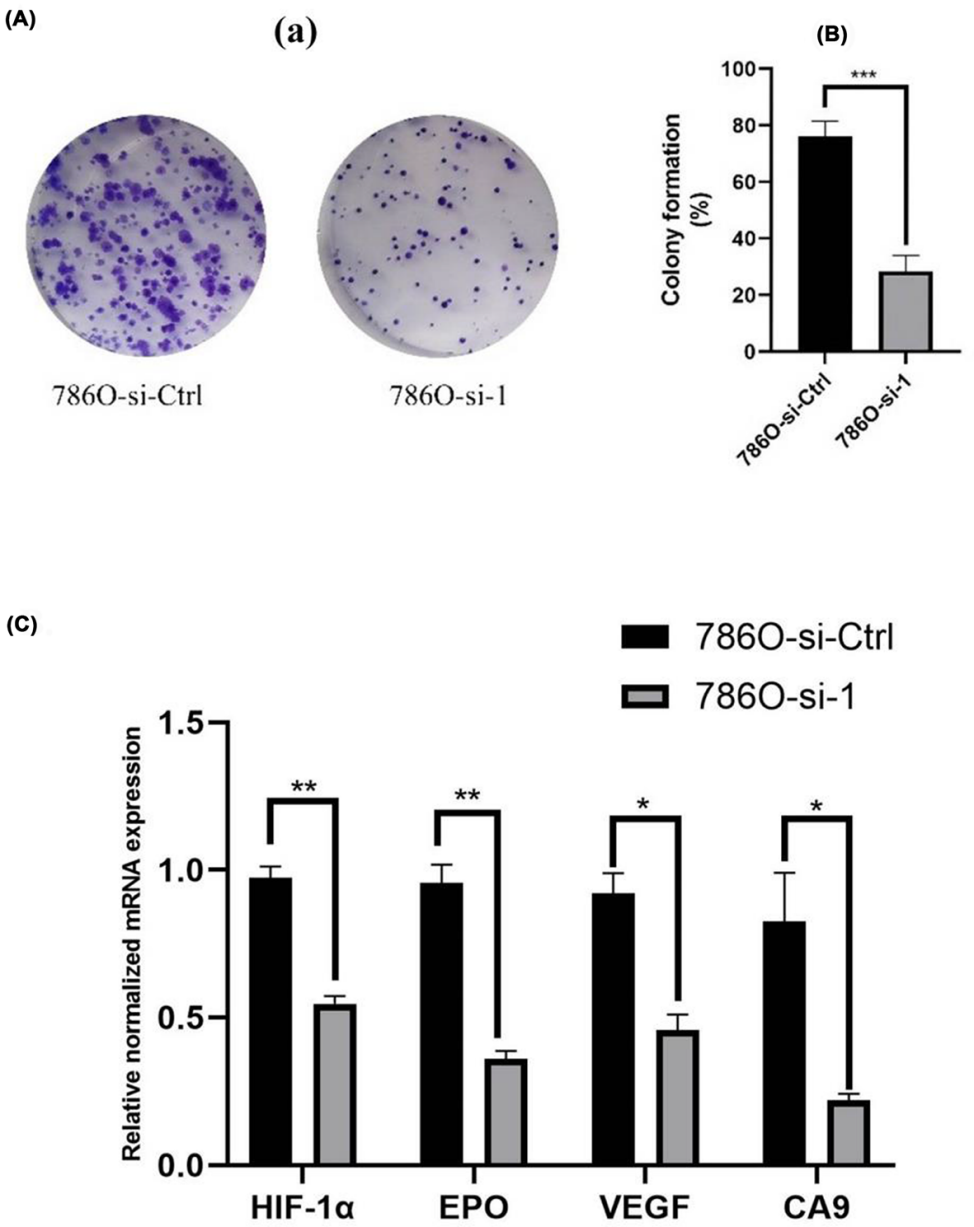
Cultured in a hypoxia condition (**A**) Knockdown of MT1X inhibits clone formation. (**B**) Knockdown of MT1X could reduce the expression of HIF-1α, EPO, VEGF, and CA9, detected by RT-qPCR (*P<0.05, **P<0.01, ***P<0.001).

We used GSEA to explore the biological functions of MT1X in ccRCC. Results were thresholded using an enrichment score (NES) >1.5 and a *P*-value < 0.05 (Table 3). Besides regulation of mast cells, MT1X was aslo found to be significantly related to cell hypoxia, which OS is an essential factor in tumor progression and distant metastasis. In addition, we detected the effect of knockdown MT1X on cell proliferation in a hypoxia condition. As our Figure 7A show, there are more clones in the 786O-si-Ctrl group than that in the 786O-si-1 group. We also add this result in part GO enrichment analysis showed that MT1X was involved in regulating and responding to metal ions, such as zinc, copper, and cadmium, which are proved to be important factors to the immune system and affect the tumor immunotherapy [25]. To gain further insight into the biological functions of MT1X, we used the human VHL defective ccRCC line 786O to detect some of the genes typically targeted by cancer therapies that are a part of the hypoxia pathway. As shown in Figure 7, knockdown of MT1X in the setting of hypoxia reduces the accumulation of HIF-1α, EPO, VEGF, and CA9, which are important factors that regulate tumor cell adaptation to hypoxia (*P*<0.05).

**Table 3:** Functional and signaling pathways correlated with MT1X expression analyzed by GSEA

Name	Size	NES	*P*-value
NAKAJIMA_MAST_CELL	19	1.84	0.004
HARRIS_HYPOXIA	38	1.80	0.020
WINTER_HYPOXIA_UP	26	1.70	0.029
ELVIDGE_HYPOXIA_UP	82	1.68	0.035
WINTER_HYPOXIA_METAGENE	98	1.66	0.035
SEMENZA_HIF1_TARGETS	20	1.64	0.046
KRIEG_HYPOXIA_VIA_KDM3A	31	1.63	0.020
GO_ZINC_ION_HOMEOSTASIS	19	1.76	0.015
GO_RESPONSE_TO_COPPER_ION	16	1.78	0.017
GO_TRANSITION_METAL_ION_HOMEOSTASIS	45	1.71	0.021
GO_RESPONSE_TO_CADMIUM_ION	22	1.81	0.026
GO_CELLULAR_RESPONSE_TO_INORGANIC_SUBSTANCE	61	1.55	0.032
GO_CELLULAR_TRANSITION_METAL_ION_HOMEOSTASIS	37	1.65	0.046

## Discussion

MTs are small cysteine-rich proteins encoded by 17 genes. Although there are few comprehensive studies on MT1s, it has been widely confirmed that MT1s have a role in homeostasis, angiogenesis, apoptosis [26], and cell differentiation [16,27]. MT1s also regulate TILs, produce immune modifiers [28], and are involved in the cell circle progression [29]. MT1s are also known to protect cells against oxidative injury, radiation, and chemotherapy. [30,31]. Another project of ours conducted the next-generation RNA sequencing analysis on the relationship between drugs and RCC, which showed that the MT family was the most closely related to the occurrence and development of RCC. So we performed bioinformatics analysis to screen a variety of factors of the MT family, such as MT-1A,-1B,-1E,-1F,-1G,-1H,-1M and -1X, and MT1X came out on top.

At the beginning of the present study, we used Oncomine and TIMER to confirm the different expression levels of MT1X between normal and tumor tissues from human cancers. Using TIMER, we found that MT1X was down-regulated by 17 human cancers and up-regulated in five types of cancer. Excepting differences in data collection and analysis methods between studies, the different expressions of MT1X in different cancers may be illustrative of the different biological properties of these tumors. In addition, we can also conclude from the results that MT1X is up-regulated in some tumors in Oncomine, while MTX is down-regulated in these tumors in TIMER. For example, MT1X is down-regulated in bladder cancer on Oncomine, but there is no difference in TIMER. In ccRCC, the results of the two databases are consistent, which also makes our subsequent study meaningful. The different expression levels of MT1X in different tumors also imply that MT1X may be involved in regulating the biological behaviors of various tumors. Besides, the differential expression of MT1X in ccRCC versus normal kidney tissues may also permit us to distinguish normal tissue from the tumor, which may be used as a detection index for tumor screening.

We confirmed that increased MT1X expression suggests poor OS in ccRCC, and we used logistic regression to correlate MT1X expression with patient clinical characteristics. Compared with T1, patients in T4 had a higher expression of MT1X. Further, MT1X expression levels were higher in patients with higher tumor grades and metastatic disease. According to our wound healing assay, knockdown of MT1X inhibited cell migration, which is consistent with the bioinformatic results that ccRCC patients with high expression of MT1X are more liable to have tumors in high tumor stage and grade and more likely to have distant metastasis. We, therefore, deduced that MT1X is related to ccRCC carcinogenesis and its malignant biologic behaviors. We further evaluated the prognostic value of MT1X in ccRCC with univariate and multivariate analyses. The multivariate Cox regression analysis showed that stage and grade are independent prognostic factors in ccRCC. MT1X can also be an independent prognostic biomarker of ccRCC. To further confirm the role of MT1X in ccRCC carcinogenesis, we knocked down MT1X in ccRCC cells 786O and found that down-regulation of MT1X induced cell apoptosis and restrained cell growth, which means MT1X may act as an oncogene in ccRCC. Furthermore, the knockdown of MT1X arrested cells in the S cell cycle. As is known, fluorouracil is an antineoplastic agent that specifically targets the S phase of the cell cycle and is the baseline treatment for ccRCC [32]. So, we speculate targeting MT1X could enhance the antitumor effect of fluorouracil. Accordingly, MT1X could be a potential biomarker that distinguishes tumor tissues from normal tissues and predict the prognosis of ccRCC.

With the development of immunotherapy cancer treatments, the immune infiltration characteristics of ccRCC are of interest. Adoptive immunotherapy may be particularly useful for patients with cancer with distant metastases. Tcm cells are superior mediators of the immunotherapy response, which is highly dependent on these cells homing to lymphoid tissues. Tcm cells were also able to increase the proliferation and persistence of cells on adoptive transfer *in vivo* [33]. Wherry [34] demonstrated that compared with Tem, Tcm has a greater capacity to persist in the host and mediate protective immunity more effectively. Using the TISIDB database, we found a positive relationship between MT1X and Tcm cells, which suggests that there is a high degree of infiltrative response by Tcm to high expression levels of MT1X. This discovery indicates that MT1X may be an efficient indicator of immunotherapy response, especially in the setting of adoptive immunotherapy for ccRCC patients with distant metastases.

According to TISIDB, high expression of MT1X significantly correlated with TGFB1, a multifunctional signaling molecule. TGFB1 can regulate immune cells and inhibit their proliferation and activation [35]. Tumor cells also seem to be more invasive and more likely to metastasize in the setting of TGFB1 overexpression [36]. Consequently, MT1X’s connection with TGFB1 may contribute to the malignant behavior of ccRCC tumor cells. CXCR4, a receptor of CXCL12, was also positively correlated with MT1X and is associated with tumor cell angiogenesis and proliferation [37]. CXCR4 is related to metastasis and lymph node spread and is associated with poor survival rates in melanoma [38] and colorectal cancer [39]. CXCR4 has also been shown to mediate different pro-metastatic events in tumor cells *in vivo*, such as invasion, migration, and extravasation [40,41]. These findings may explain the association between MT1X expression and a poor prognosis.

Another meaningful aspect of this study is the correlation between MT1X and hypoxia. A pivotal characteristic of tumor cells is their adaptation to a hypoxic environment mediated by hypoxia-inducible factors (HIFs) [42]. A hypoxic microenvironment influences tumor cell invasiveness, metastatic properties, metabolic adaptations, and stemness, leading to the development of aggressive neoplasms [43]. Von Hippel–Lindau (VHL) is a tumor suppressor whose loss is frequently seen in ccRCC patients [44]. Loss of VHL leads to the accumulation of HIF-α, which is a hypoxia-inducible factor. The loss of VHL also results in activating HIF target factors such as EPO, VEGF, and HO-1, which facilitate the angiogenesis, proliferation, and metastasis of ccRCC [45]. Our colon formation assay and RT-qPCR results show that knockdown of MT1X could inhibit the cell proliferation and reduced the accumulation of hypoxia-related factors, thus illustrating MT1X’s oncogene role in ccRCC.

Exposure to metals and solvents is nephron-carcinogenic [46]. The relationship between cadmium and renal cancer was proposed over 40 years ago [47]. Lead toxicity has also been shown to promote renal tumor formation in a large number of studies, and higher lead levels in the blood are linked with RCC [48,49]. Cadmium and lead induce phenotypic changes related to RCC cell aggregation, migration, and invasion [50]. GSEA analysis in our study showed that MT1X is notably involved in both hypoxia and metal ion homeostasis regulation, which are essential factors in the occurrence and progression of ccRCC.

In conclusion, this is the first study to describe the independent prognostic value of MT1X in ccRCC and identify MT1X as a new potential biomarker of ccRCC. Our work may also be useful for understanding the interactions between MT1X and the immune system in ccRCC and for defining the underlying pro-carcinogenic mechanisms for MT1X in ccRCC. Our cellular experiments demonstrated the oncogenic role of MT1X in ccRCC cells in the context of bioinformatic results. Further studies on the biological impact of MT1X and more clinical data on MT1X and ccRCC are strongly recommended.

## Supplementary Material

Supplementary Tables S1-S2

## Data Availability

All datasets generated for this study are included in the manuscript.

## Abbreviations

ccRCC: clear cell RCC
EGA: European Genome-Phenome Archive
GEO: Gene Expression Omnibus
GSEA: gene set enrichment analysis
HIF: hypoxia-inducible factor
M0: patients without metastasis
M1: patiens with metastasis
MT1: metallothionein 1
OR: odds ratio
OS: overall survival
PFA: paraformaldehyde
PI: propidium iodide
q-PCR: quantitative PCR
RCC: renal cell carcinoma
TCGA: The Cancer Genome Atlas Program
TIL: tumor-infiltrating lymphocyte
VHL: Von Hippel–Lindau
