# Supplementary material for: MT1X is an oncogene and indicates prognosis in ccRCC
Source: Biosci Rep. 2022 Oct 18;42(10):BSR20221128. doi: 10.1042/BSR20221128 (PMC9583764; doi:10.1042/BSR20221128)
Supplement: Supplementary Tables S1-S2 [file BSR-2022-1128_supp.pdf]

Supplementary Table 1. Target sequence of si-MT1X

name	sense	antisense
si-MT1X-1	GGCAAUAAAUUCAUCUAGACUdTdT	AGUCUAGAUGAAUUUAUUGCCdTdT
si-MT1X-2	GCUGUGCUCUCAGAUGUAAAUdTdT	AUUUACAUCUGAGAGCACAGCdTdT

Supplementary Table 2. Primers for mRNA used in this study.

Primer name	Forward	Reverse
MT1X	GCTCGCCTGTTGGCTCCTG	TGCAGATGCAGCCCTGGGC
HIF-1α	AAGAACTTTTAGGCCGCTCA	CAACCCAGACATATCCACCTC
VEGF	TGCCAAGTGGTCCCAG	GTGAGGTTTGATCCGC
EPO	AACAATCACTGCTGACACTT	AGTTGCTCTCTGGACAGT
CA9	TATGAGGGGTCTCTGACTACA	TTCTCATCTGCACAAGGAAC
GAPDH	GGAGCGAGATCCCTCCAAAAT	GGCTGTTGTCATACTTCTCATGG
